# Safety and Efficiency of Robotic Versus Laparoscopic Revisional Bariatric Surgery: A Systematic Review and Meta-Analysis

**DOI:** 10.3390/medsci14060623

**Published:** 2026-09-30

**Authors:** Hareesha Rishab Bharadwaj, Ethan Danda, Syed Hasham Ali, Priyal Dalal, Venkata Sunkesula, Ashish Sharma, Arkadeep Dhali, Rahman Hameed Mohammed Abdul, Fariha Hasan, Abdullah Sultany, Farhan Gohar, Dushyant Singh Dahiya

**Affiliations:** 1 Royal Stoke University Hospital, University Hospitals of North Midlands NHS Trust, Stoke-on-Trent ST4 6QG, UK; rishabbharadwaj31@gmail.com (H.R.B.); ethandanda@gmail.com (E.D.); doctorgohar@gmail.com (F.G.); 2 Immediate Care Plus, East Dundee, IL 60118, USA; syedhashamali00@gmail.com; 3 School of Medicine, University of Lancashire, Preston PR1 2HE, UK; priyaldalal15@gmail.com; 4 Division of Gastroenterology and Hepatology, Case Western Reserve University/The MetroHealth System, Cleveland, OH 44109, USA; kumarsvc@gmail.com; 5 Department of Medicine, Yale New Haven Hospital, New Haven, CT 06510, USA; ashish.sha688@gmail.com; 6 Academic Unit of Gastroenterology, Sheffield Teaching Hospitals NHS Foundation Trust, Sheffield S5 7AU, UK; 7 School of Medicine, Dentistry and Biomedical Sciences, Queen’s University Belfast, Belfast BT7 1NN, UK; 8 Department of Gastroenterology, King’s Mill Hospital, Sutton-in-Ashfield NG17 4JL, UK; mdabdulrahman2112@gmail.com; 9 Department of Gastroenterology & Hepatology, Cooper University Hospital, Camden, NJ 08103, USA; farihahassan100@gmail.com; 10 Department of Internal Medicine, Guthrie Robert Packer Hospital, Sayre, PA 18840, USA; abdullahsultany142160@gmail.com; 11 Department of Gastroenterology & Hepatology, Turkey Creek Medical Center, Tennova Healthcare, Knoxville, TN 37934, USA; dush.dahiya@gmail.com

**Keywords:** bariatric surgery, revisional surgery, general surgery, gastrointestinal sciences

## Abstract

**Background:** Revisional bariatric surgery is increasingly performed due to weight regain, inadequate weight loss, or complications following primary bariatric procedures. These operations are technically challenging, and while laparoscopic surgery remains the standard approach, robotic platforms have gained adoption. However, comparative evidence on safety and efficiency remains limited and methodologically heterogeneous. **Methods:** A systematic review and meta-analysis was conducted in accordance with PRISMA guidelines. PubMed/Medline, Embase, Scopus, Web of Science, and Cochrane Central were searched. Comparative studies of robotic versus laparoscopic revisional bariatric surgery were included. To reduce bias from overlapping cohorts, we systematically identified and excluded duplicate datasets from national and institutional registries, prioritising the most contemporary and comprehensive reports, and retained partially overlapping studies only when the non-overlapping period spanned ≥3 years. Study quality was assessed using the Newcastle–Ottawa Scale. Outcomes analysed included length of hospital stay, operative time, postoperative complications, reoperation, anastomotic leakage and pneumonia. Random-effects models were used to generate pooled estimates. **Results:** Six retrospective studies encompassing 7199 robotic and 34,584 laparoscopic procedures were included. Robotic surgery was associated with a statistically significant reduction in length of hospital stay compared with laparoscopy (mean difference −1.06 days; 95% CI −1.97 to −0.15; I^2^ = 84%). This finding was sensitive to the inclusion of a single large registry-based study. No significant differences were observed in the overall postoperative complications, reoperation rates, anastomotic leakage or pneumonia. Operative time did not differ significantly in the primary analysis. (I^2^ = 93%), but sensitivity analyses suggested shorter procedure durations with laparoscopic surgery after exclusion of influential studies. **Conclusions:** In this systematic review and meta-analysis, robotic revisional bariatric surgery was associated with a shorter length of hospital stay and similar short-term safety outcomes to laparoscopic surgery. However, the very high statistical heterogeneity, observational design and potential for selection bias mean that these findings should be interpreted as hypothesis-generating rather than definitive evidence of superiority or equivalence. Larger, prospective, multi-centre studies with standardised outcome definitions and robust adjustment for confounding are needed to clarify the comparative clinical and cost-effectiveness of robotic versus laparoscopic approaches in revisional bariatric surgery.

## 1. Introduction

The last couple of decades have seen a dramatic rise in obesity prevalence globally [1], with the global economic impact of overweight and obesity projected to reach approximately US$3.3 trillion by 2030 and US$4.3 trillion annually by 2035, underscoring obesity as a major global public health priority [2]. Bariatric surgery has emerged as a safe, effective, and durable treatment option for obesity and its associated health consequences. Consequently, demand for bariatric surgery has increased substantially, accompanied by a corresponding rise in revisional procedures [3]. In Australia, bariatric surgery rates increased by 89.7% between 2013 and 2022 [4], while in the United States, annual utilisation increased from 59.2 to 79.6 per 100,000 adults between 2012 and 2018 [5]. The increasing volume of primary bariatric procedures has consequently resulted in more patients requiring revision, primarily performed for weight regain, inadequate weight loss, or complications from the index operation [6]. Revisional bariatric surgery is most commonly undertaken because of inadequate weight loss or weight regain, although complications and procedure-specific indications also contribute to the need for revision [7].

Revisional bariatric surgery is technically demanding because previous operations may result in dense adhesions, distorted anatomy, altered tissue planes, and tissue friability, increasing operative complexity and the potential risk of complications compared with primary procedures [8]. Laparoscopic surgery has traditionally remained the standard minimally invasive approach for revisional bariatric procedures, with established safety and efficacy [9]. However, robotic platforms have increasingly been adopted because of their enhanced instrument articulation, three-dimensional high-definition visualisation, and improved surgeon ergonomics [10,11]. These characteristics may be particularly advantageous in the revisional setting, where precise dissection through scarred and distorted anatomical planes is often required.

Despite increasing adoption of robotic surgery, the comparative safety and perioperative efficiency of robotic versus laparoscopic revisional bariatric surgery remain uncertain [12,13,14]. The available evidence consists predominantly of retrospective studies with heterogeneous patient populations, procedural indications, institutional practices, and levels of surgical experience. Differences in surgeon expertise, institutional case volume, patient selection, and robotic learning curves may further influence observed outcomes. Previous meta-analyses have attempted to synthesise this evidence but have been limited by methodological concerns, including potential overlap between cohorts derived from national and institutional databases and variable approaches to assessing study quality [13,14]. Moreover, additional comparative evidence, including large contemporary registry-based analyses, has become available since earlier syntheses, providing an opportunity to reassess the comparative outcomes of these approaches.

The present systematic review and meta-analysis therefore aims to update the existing evidence while addressing several methodological limitations of previous reviews. First, we systematically assessed studies originating from national and institutional databases for overlapping patient cohorts and excluded duplicate datasets, prioritising the most contemporary and comprehensive analysis when substantial overlap was identified. Second, we prespecified a conservative rule for partial cohort overlap, retaining partially overlapping studies only when at least three years of the study period were non-overlapping, thereby ensuring a substantial proportion of potentially unique patients. Third, we restricted inclusion to comparative studies with extractable robotic and laparoscopic outcome data and performed a structured Newcastle–Ottawa Scale assessment, incorporating study quality and the extent of adjustment for confounding into interpretation of the pooled findings. These methodological refinements were intended to reduce duplication, minimise selection bias, and provide a more reliable synthesis of the contemporary evidence. The primary outcomes were length of hospital stay, operative time, postoperative complications, and reoperation, with anastomotic leakage and pneumonia also evaluated where reported.

## 2. Methods

This meta-analysis was performed in accordance with the Preferred Reporting Items for Systematic Reviews and Meta-Analyses (PRISMA) guidelines [15]. As it utilized data from previously published studies, institutional review board (IRB) approval and patient informed consent were not required. This Systematic Review has not been registered on platforms such as PROSPERO.

### 2.1. Eligibility Criteria

Only original studies were included where they: (1) reported outcomes for patients undergoing revisional bariatric surgery following a primary bariatric procedure, and (2) allowed for comparison of robotic versus laparoscopic approaches. Studies were excluded if they: (1) Only included unrelated procedures (e.g., laparotomy or non-bariatric surgery), (2) were systematic reviews, meta-analyses, commentaries, abstracts, or case reports, or (3) involved non-human subjects.

#### Risk of Bias Adjustment

In cases where multiple studies utilised the same patient databases with overlapping study periods, we prioritised the most contemporary and comprehensive dataset to preserve data integrity, minimise duplication and limit bias. Complete temporal overlap resulted in the exclusion of the earlier or small study. Partial study overlap was considered acceptable where the non-overlapping period spanned greater than three years. This threshold was chosen as a priori to ensure that at least half of a typical 5–6-year study period would comprise unique patients, thereby materially reducing the risk that pooled estimates are driven by repeated inclusion of the same individuals. This conservative approach is more stringent than that employed in prior meta-analyses and was intended to limit dataset overlap that has not been adequately addressed previously.

### 2.2. Literature Search Process

PubMed/Medline, Cochrane Central, Embase, Scopus, and Web of Science were searched from database inception and the literature search was concluded on 12 December 2024; all subsequent activities (data extraction, analysis and manuscript preparation) were performed after this date. The search strategy employed relevant keywords and MeSH terms, including but not limited to “robotic”, “laparoscopic”, “bariatric” and “DaVinci” with various iterations of these terms. Full search strategies are provided in Supplementary Table S1. The search did not apply any filters, language or time exclusions.

Two reviewers (R.H.B. and S.H.A.) independently screened titles, abstracts and full texts for study eligibility. Prior to screening, duplicates were removed in EndNote. Reference lists were manually searched for additional studies. Any discrepancies were resolved by a third reviewer (T.M.).

### 2.3. Outcomes of Interest, Data Extraction, and Risk of Bias Assessment

The following outcomes were selected for analysis (1) length of hospital stay (days), (2) operative time (minutes), (3) incidence of reoperation, (4) incidence of postoperative complications, (5) incidence of leakage, and (6) incidence of pneumonia.

Data was extracted to a standardised Microsoft Excel template. Two independent reviewers (S.H.A and R.H.B) conducted risk of bias assessments using the Newcastle-Ottawa scale for observational studies, with inconsistencies resolved through discussion [16].

### 2.4. Statistical Analysis

A two-arm meta-analysis was performed using Review Manager version 5.4.1 (The Cochrane Collaboration, The Nordic Cochrane Centre, Copenhagen, Denmark). A random-effects model was applied to account for any heterogeneity in outcome definitions, study methodologies, and procedures.

For all dichotomous outcomes, risk ratios (RR) and corresponding 95% confidence intervals (CI) were calculated from pooled data under the inverse-variance method, with results presented as robotic versus laparoscopic approach. For continuous outcomes, means and standard deviations were extracted, with corresponding mean differences (MD) and 95% CIs calculated using the inverse-variance method, pooled, and analysed. In cases where data was presented as medians with interquartile ranges (IQR), values were imputed to means and SDs using methods described by Wan et al.’s [17].

Where studies stratified bariatric procedure by type (e.g., Roux en Y, sleeve gastrectomy), values were averaged for continuous outcomes and summed for dichotomous outcomes. For studies reporting procedure-specific odds ratios (ORs) rather than pooled estimates, ORs were combined using the inverse-variance method applied on the log-transformed ORs. Reported ORs and 95% confidence intervals were log-transformed, standard errors were derived from the confidence limits, and a weighted average was calculated. Pooled estimates were then converted back to the OR scale, with 95% confidence intervals. Propensity-weighted or otherwise adjusted data was used where available.

Statistical significance was defined as a *p*-value < 0.05. A leave one-out-sensitivity check was employed for all outcomes to assess robustness. Forest plots were generated to visually represent the results. The Higgins (I^2^) statistic was used to quantify heterogeneity with 25–50% considered mild, 50–75% as moderate, and >75% as severe heterogeneity. Heterogeneity was investigated when I^2^ exceeded 40% in statistically significant outcomes by conducting sensitivity analysis to identify outliers and subgroup analysis if necessary. A robust subgroup analysis would necessitate at least 3 studies per subgroup and if that threshold was not met, said analysis was not undertaken as directed by the Cochrane Handbook [18]. Publication bias and meta-regression were planned only if >10 studies reported an outcome.

## 3. Results

### 3.1. Literature Search Synthesis

**Figure 1:**
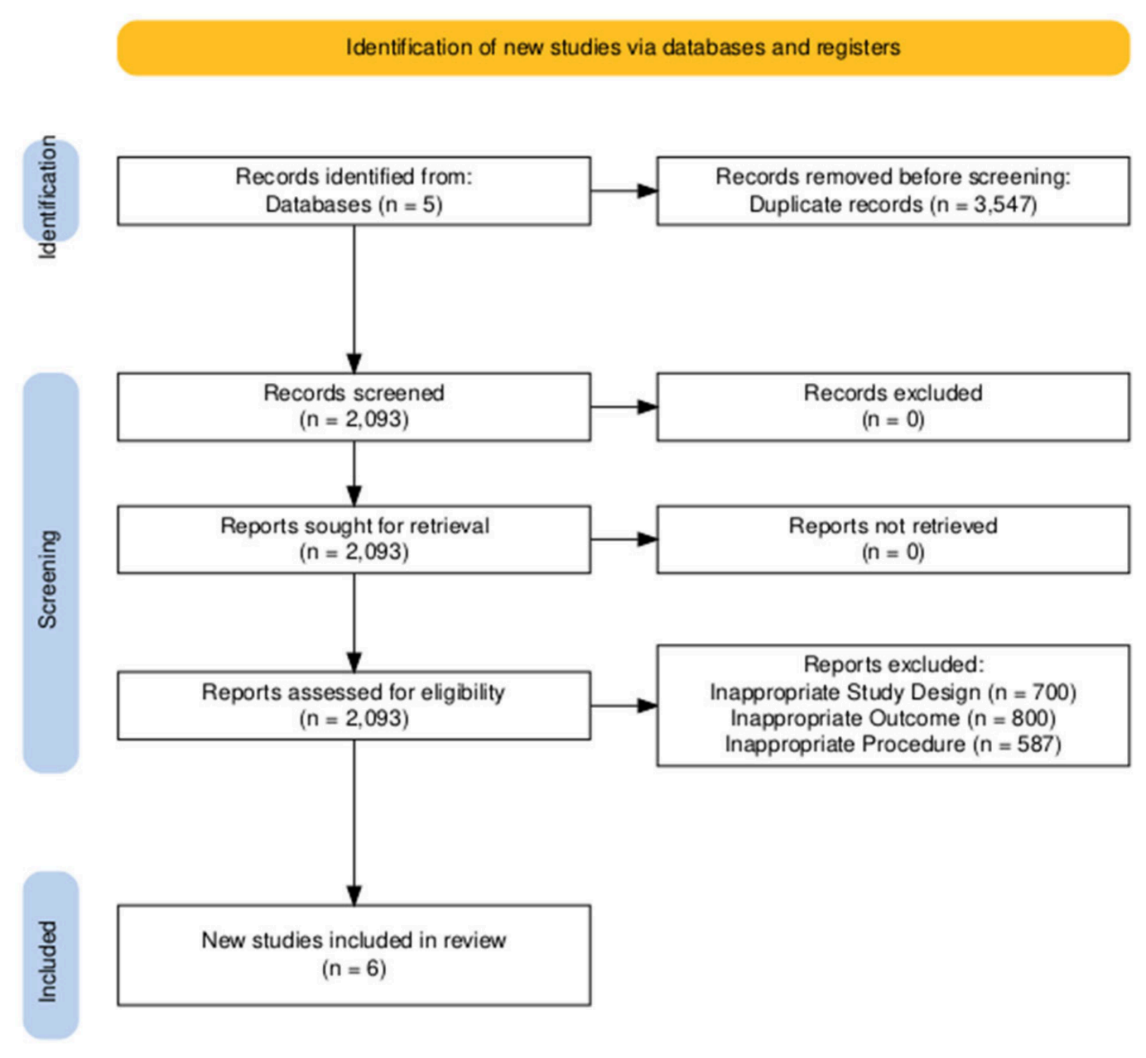
PRISMA Flowchart.

The search strategy employed across PubMed/Medline, Cochrane Central, Embase, Scopus, and Web of Science databases identified 2093 unique records, of which 6 studies met our inclusion criteria [19,20,21,22,23,24]. In contrast to existing literature, only the most recent and comprehensive analysis of the Metabolic and Bariatric Surgery Accreditation and Quality Improvement Program (MBSAQIP) database was included, as other analyses of the database were considered subsets [21]. Additionally, individual hospital studies contributing data to the MBSAQIP were excluded. Gray et al. 2018 was an exception to this and was retained as its data collection began in 2007, predating the 2012 data inclusion threshold of the included MBSAQIP analysis by ≥3 years [22]. This decision was consistent with our prespecified rule that partial overlap is acceptable only when the non-overlapping period spans at least three years, ensuring a substantial proportion of unique patients. Elchaar et al. 2020 was included only for length of procedure outcomes, not otherwise reported in the primary MBSAQIP study [20,21]. The complete study selection process is graphically outlined in the PRISMA flowchart (Figure 1).

### 3.2. Study Characteristics

All 6 included studies were retrospective, published between 2013–2024 and spanned patient data from 2000–2022. Most studies were conducted in the United States, with additional data from Germany, Switzerland and Italy. While inclusion criteria varied, they were largely comparable. A full summary of study characteristics has been tabulated in Table 1.

### 3.3. Risk of Bias Assessment

All studies were evaluated using the Newcastle-Ottawa Scale, given their retrospective design, with most studies scoring low on comparability due to a lack of reported propensity-weighted or adjusted data. However, all studies included were otherwise well conducted and achieved a minimum score of 7. The full results stratified by score per domain are presented in Supplementary Table S2.

### 3.4. Baseline Characteristics

The meta-analysis had a preponderance of women, with most procedures being Roux en Y, followed by sleeve gastrectomies. Patient age ranged from 44.0 to 50.7 years old, with no individual study reporting any significant differences in the ages of the laparoscopic versus robotic surgery cohort. A full summary of baseline characteristics has been tabulated in Table 2.

### 3.5. Results of the Meta-Analysis

#### 3.5.1. Length of Stay (Days)

**Figure 2:**
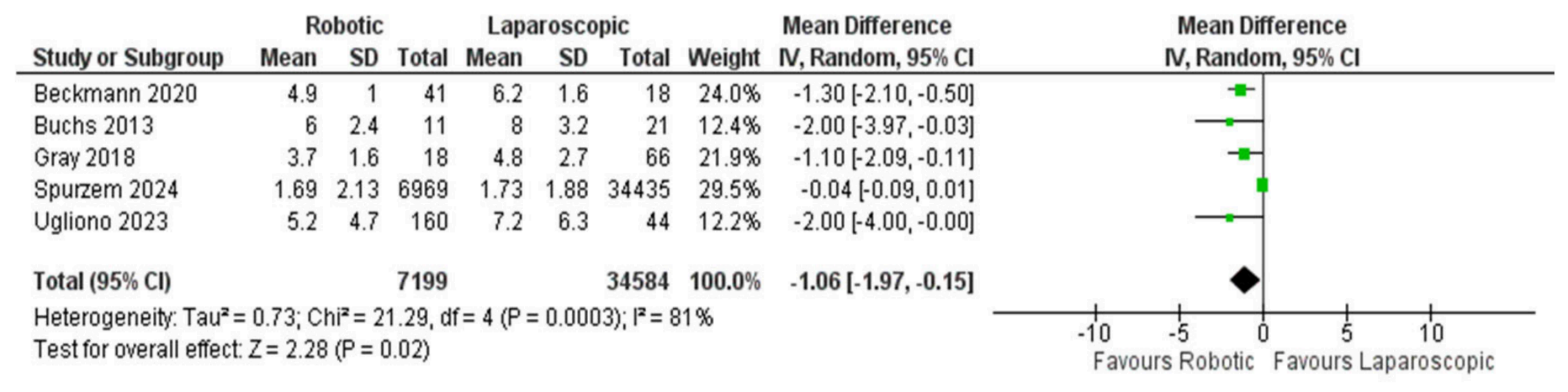
Forest plot showing mean difference in length of stay (days) between robotic and laparoscopic groups on pooled analysis of 5 studies [19,21,22,23,24].

A pooled analysis of five studies [19,21,22,23,24], including 7199 robotic and 34,584 laparoscopic procedures, demonstrated that robotic revisional bariatric surgery was associated with a statistically significant reduction in length of hospital stay compared to laparoscopic surgery (MD −1.06, 95% CI −1.97 to −0.15, *p* = 0.02, I^2^ = 84%) (Figure 2). Sensitivity analysis identified Spurzem et al. as an outlier, exclusion of which eliminated heterogeneity (I^2^ = 0%) while preserving the direction and significance of the effect [21].

Given the very high between study heterogeneity and the observational nature of the included data, this finding should be interpeted as hypothesis-generating rather than definitive evidence of a robust benefit, and may reflect differences in a patient selection, perioperative pathways and discharge criteria across centres.

#### 3.5.2. Length of Procedure (Minutes)

**Figure 3:**
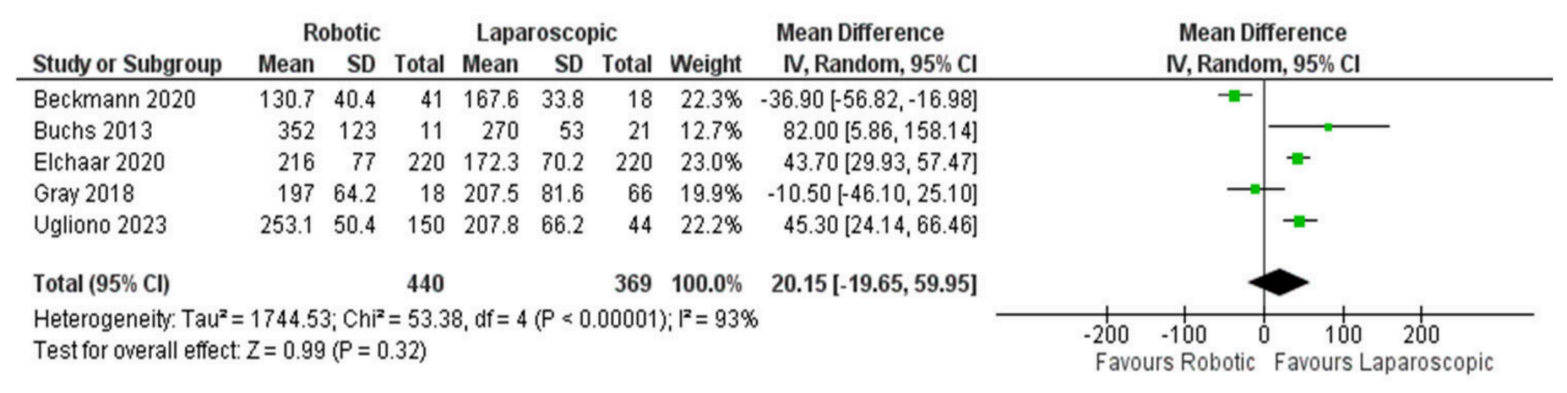
Forest plot showing mean difference in length of procedure (minutes) between robotic and laparoscopic groups in a pooled analysis of 5 studies [19,20,22,23,24].

Five studies reported length of procedure [19,20,22,23,24]. Overall, there was no significant mean difference between robotic and laparoscopic approaches (mean difference 20.15, 95% CI: −19.65 to 59.95, *p* = 0.32, I2 =93%) (Figure 3). Sensitivity analysis revealed significant heterogeneity. Exclusion of Beckmann et al. resulted in a statistically significant difference favouring laparoscopic surgeries (MD 35.30, 95% CI: 10.81 to 59.97, *p* = 0.005, I^2^ = 68%) [24]. Subsequent exclusion of Gray et al. reduced the heterogeneity to 0% and strengthened the association, with laparoscopic surgery demonstrating shorter procedure lengths (MD 45.03, 95% CI: 33.62 to 56.44, *p* < 0.00001, I^2^ = 0%) [22].

These findings indicate that the pooled estimate for operative time is highly sensitive to individual studies and underlying clinical heterogeneity, and should therefore be interpreted with caution.

#### 3.5.3. Total Postoperative Complications

**Figure 4:**
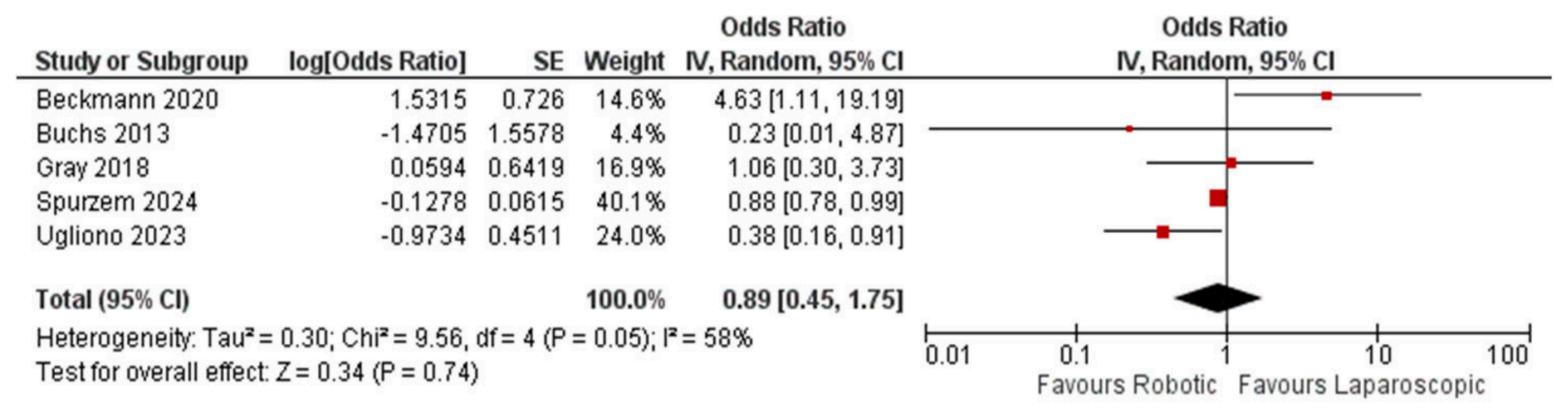
Forest plot showing odds ratio for total postoperative complications between robotic and laparoscopic groups in a pooled analysis of 5 studies [19,21,22,23,24].

Pooled analysis of five studies found no significant differences in total postoperative complications between robotic and laparoscopic revisional surgery (OR 0.89, 95% CI 0.45 to 1.75, *p* = 0.74, I^2^ = 58%) [19,21,22,23,24] (Figure 4). Sensitivity analysis did not change the direction or significance of this result. The absence of significant differences does not demonstrate the equivalence or non-inferiority of the two approaches.

#### 3.5.4. Reoperation

**Figure 5:**
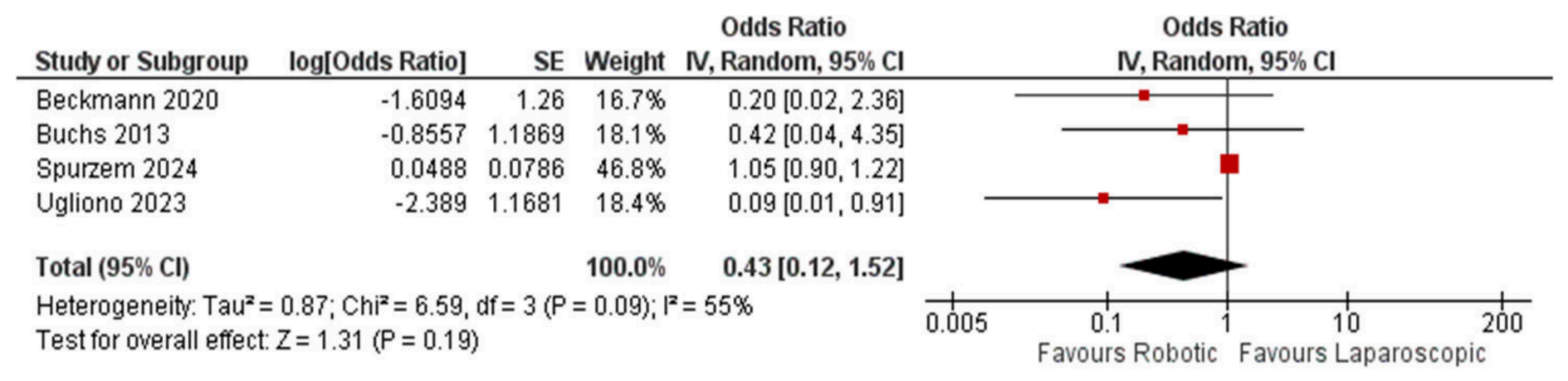
Forest plot showing odds ratio for reoperation rates between robotic and laparoscopic groups in a pooled analysis of 4 studies [19,21,23,24].

Four studies reported reoperation rates [19,21,23,24], showing no significant difference between groups (OR 0.43, 95% CI 0.12 to 1.52, *p* = 0.19, I^2^ = 55%) (Figure 5). Sensitivity analysis identified exclusion of Spurzem et al. shifted the effect direction and significance, with statistically fewer reoperations with robotic surgery (OR 0.20, 95% CI 0.05 to 0.77, *p* = 0.02, I^2^ = 0%) [21]. The absence of significant differences does not demonstrate the equivalence or non-inferiority of the two approaches.

#### 3.5.5. Leakage

**Figure 6:**
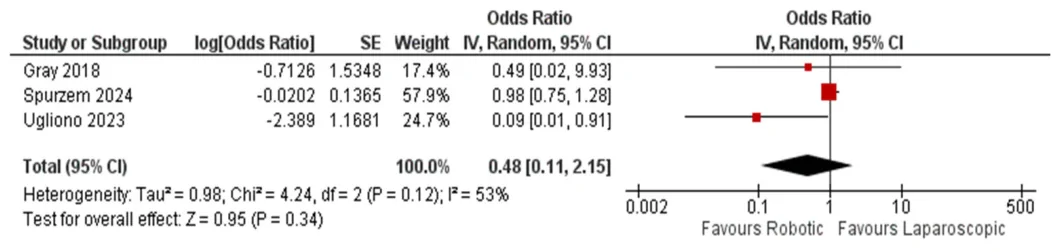
Forest plot showing odds ratio for leakage rates between robotic and laparoscopic groups in a pooled analysis of 3 studies [21,22,23].

Pooled analysis of three studies [21,22,23] found the approach was not significantly associated with differences in leakage rate (OR 0.48, 95% CI 0.11 to 2.15, *p* = 0.34, I^2^ = 53%) (Figure 6). Sensitivity analysis was not performed due to limited studies. The absence of significant differences does not demonstrate the equivalence or non-inferiority of the two approaches.

#### 3.5.6. Pneumonia

**Figure 7:**
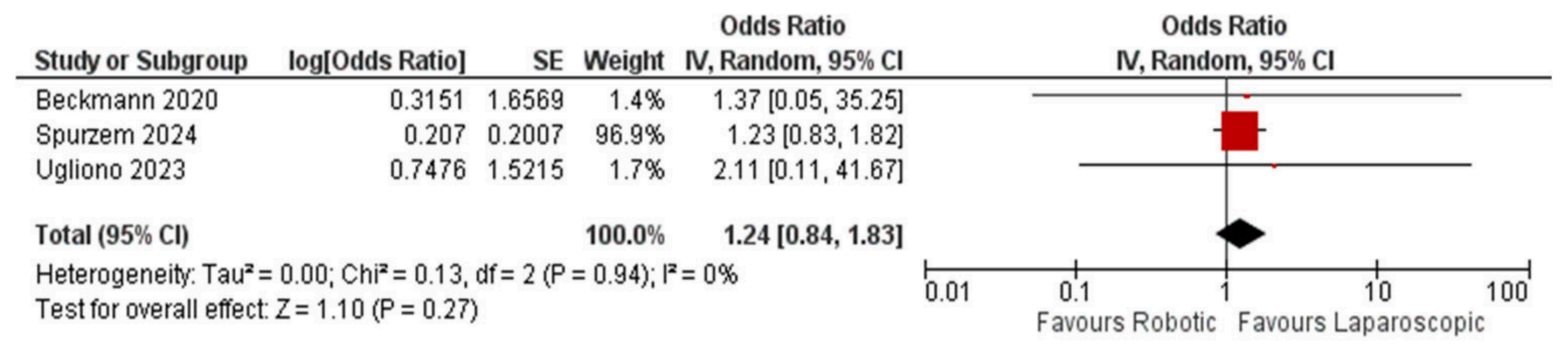
Forest plot showing odds ratio for pneumonia between robotic and laparoscopic groups in a pooled analysis of 3 studies [21,23,24].

Pooled analysis of three studies demonstrated no statistically significant difference in pneumonia rates between robotic and laparoscopic revisional bariatric surgery (OR 1.24, 95% CI 0.84 to 1.83, *p* = 0.27, I^2^ = 0%) (Figure 7) [21,23,24]. Due to the paucity of studies, a sensitivity analysis was not conducted. The absence of significant differences does not demonstrate the equivalence or non-inferiority of the two approaches.

## 4. Discussion

This systematic review and meta-analysis examined the comparative safety and efficiency of robotic versus laparoscopic revisional bariatric surgery using evidence spanning approximately 25 years. Across six retrospective comparative studies involving 7199 robotic and 34,584 laparoscopic procedures, robotic surgery was associated with a statistically significant reduction in length of hospital stay, while no statistically significant differences were observed in total postoperative complications, reoperation, anastomotic leakage, or pneumonia. Operative time did not differ significantly between approaches in the primary analysis. However, the interpretation of these findings requires considerable caution because the available evidence is entirely retrospective, the included studies were clinically heterogeneous, and the results for both length of stay and operative time demonstrated substantial sensitivity to individual studies. Accordingly, the findings should be regarded as hypothesis-generating rather than definitive evidence of superiority or equivalence between robotic and laparoscopic approaches.

The present review was undertaken in the context of previous meta-analyses that have reported broadly similar perioperative outcomes but have been limited by methodological concerns, particularly potential overlap between patient cohorts and variable assessment of study quality [13,14]. An important strength of the current review was therefore the systematic assessment of potential cohort duplication across national and institutional databases. Where overlapping datasets were identified, the most contemporary and comprehensive analysis was prioritised, while studies with partial overlap were retained only where at least three years of the study period were non-overlapping. This approach was intended to reduce the possibility that the same patients would contribute repeatedly to pooled estimates and thereby artificially increase the apparent precision of the findings. We also restricted inclusion to comparative studies with extractable robotic and laparoscopic data and performed a structured Newcastle–Ottawa assessment of study quality. These methodological measures distinguish the present synthesis from earlier reviews and provide a more carefully controlled assessment of the contemporary evidence base.

The statistically significant reduction in length of hospital stay associated with robotic surgery is an interesting finding, but should not be interpreted as definitive evidence that the robotic approach intrinsically produces faster postoperative recovery. Although the pooled analysis demonstrated a mean reduction of 1.06 days, heterogeneity was very high (I^2^ = 84%). Furthermore, the sensitivity analysis demonstrated that exclusion of the large registry-based study by Spurzem et al. eliminated the statistical heterogeneity while preserving the direction and significance of the association [21]. The dependence of the pooled estimate on a single large study indicates that the magnitude of the observed effect may be influenced substantially by characteristics of the underlying dataset rather than solely by the operative platform.

Several factors may contribute to this heterogeneity. Revisional bariatric surgery encompasses a diverse range of procedures and indications, from relatively straightforward revisions following gastric banding to technically complex conversions or revisions following sleeve gastrectomy and Roux-en-Y gastric bypass. Differences in operative complexity, the distribution of index procedures, surgeon experience, institutional case volume, perioperative management, postoperative monitoring, and discharge criteria may therefore substantially influence length of stay. Differences between healthcare systems and institutional pathways may also contribute to variation in discharge practices. Consequently, a shorter hospital stay observed in one healthcare setting may not necessarily be reproduced in another. The apparent association between robotic surgery and reduced hospitalisation may therefore reflect, at least in part, centre-specific pathways and patient selection rather than a direct causal effect of the robotic platform.

The possibility of selection bias is particularly important when interpreting the length-of-stay finding. All included studies were retrospective, and only two of the six studies provided propensity score-matched or otherwise adjusted data. The absence of consistent adjustment for baseline characteristics limits the ability to determine whether differences in outcomes are attributable to the surgical approach or to systematic differences between patients selected for robotic and laparoscopic surgery. In particular, baseline BMI differed between treatment groups, with the laparoscopic cohort having a higher mean BMI than the robotic cohort in the available data. Because higher BMI may be associated with greater operative complexity and perioperative risk, this imbalance could contribute to longer hospitalisation in the laparoscopic group independently of surgical approach. It is therefore plausible that some of the observed reduction in length of stay reflects differences in patient selection rather than a direct benefit of robotic surgery.

Other potential confounders were also incompletely reported across the included studies. Surgeon experience with robotic and laparoscopic techniques, institutional robotic case volume, the stage of the institutional learning curve, and the specific indication and type of revisional procedure may all influence perioperative outcomes. These factors are particularly relevant because robotic surgery is often preferentially adopted by high-volume centres or surgeons with specific expertise in complex minimally invasive procedures. Conversely, the technology may initially be associated with longer operative times during the learning phase. Because these characteristics were not consistently reported or adjusted for, causal inferences regarding the superiority of either approach cannot be reliably made.

With respect to safety outcomes, no statistically significant differences were identified between robotic and laparoscopic approaches for total postoperative complications, reoperation, anastomotic leakage, or pneumonia. These findings are broadly consistent with previous literature suggesting that robotic surgery can be performed in complex revisional bariatric cases without an evident increase in short-term morbidity [13,14]. However, the absence of a statistically significant difference should not be interpreted as proof of equivalence or non-inferiority. The relatively small number of studies, limited number of events for some outcomes, and observational nature of the available evidence reduce the certainty with which differences between approaches can be excluded. Accordingly, the present findings indicate that no statistically significant increase in these complications was detected, rather than demonstrating that the two approaches have identical safety profiles.

Operative time represents a particularly important area of uncertainty. In the primary analysis, there was no statistically significant difference between robotic and laparoscopic surgery, but heterogeneity was extremely high (I^2^ = 93%). More importantly, the direction and magnitude of the pooled estimate changed substantially during sensitivity analysis. Exclusion of Beckmann et al. resulted in a statistically significant difference favouring laparoscopic surgery, with a mean difference of 35.30 min and residual heterogeneity of 68% [24]. Subsequent exclusion of Gray et al. reduced heterogeneity to 0% and further increased the magnitude of the difference, with laparoscopic surgery demonstrating a 45.03-min shorter procedure duration [22]. These findings demonstrate that the pooled operative-time estimate is highly sensitive to individual studies and should therefore not be interpreted as evidence that the two approaches have definitively equivalent operative efficiency [25,26,27,28,29,30,31].

The substantial heterogeneity in operative time is likely multifactorial. Differences in the complexity and type of revisional procedures, surgeon experience, institutional case volume, robotic learning curves, docking and setup times, and perioperative workflows may all influence procedure duration [32,33]. The temporal evolution of robotic technology and increasing operator familiarity may also contribute to differences between studies conducted at different time points. Previous evidence suggests that operative efficiency may improve with increasing surgical experience and case volume [10,11,32,33]. Therefore, operative-time comparisons derived from heterogeneous retrospective cohorts spanning different eras and institutions may not accurately reflect the operative efficiency achievable with contemporary robotic practice.

The very high heterogeneity observed for both length of stay and operative time is an important limitation of the current evidence base. Although a random-effects model was used to account for between-study variation, statistical modelling cannot resolve underlying clinical or methodological heterogeneity. In particular, the six included studies differed in study period, patient population, database source, revisional procedure, institutional setting, and reporting practices. The small number of available studies also prevented meaningful subgroup analyses according to index procedure, type of revision, centre volume, or surgeon experience. These factors are clinically important because a pooled estimate across different revisional pathways may obscure meaningful differences between specific procedures. Future studies should therefore report outcomes separately for distinct revisional pathways, such as band-to-bypass conversion, sleeve-to-bypass conversion, and revision of previous gastric bypass, wherever possible.

The methodological quality of the included evidence further limits the strength of the conclusions. All six studies were retrospective, and most received lower Newcastle–Ottawa comparability scores because of limited reporting of propensity-matched or adjusted analyses. Although all studies achieved a minimum Newcastle–Ottawa score of 7 and were otherwise considered reasonably well conducted, quality assessment does not eliminate the risk of residual confounding inherent to observational research. In particular, differences in patient selection, BMI, comorbidity burden, surgeon expertise, centre volume, and procedure complexity may remain incompletely controlled. The fact that only two studies incorporated propensity score matching or other adjustment reinforces the need to avoid causal interpretations of the pooled associations.

The present review nevertheless has several strengths. It provides an updated synthesis of comparative evidence while specifically addressing potential cohort overlap, a methodological issue that may otherwise result in repeated inclusion of the same patients and overrepresentation of large databases. The prespecified approach to partial overlap, requiring a substantial non-overlapping period, further reduced the risk of duplicate patient inclusion. In addition, the structured Newcastle–Ottawa assessment allowed study quality and the extent of adjustment for confounding to be considered when interpreting the findings. The inclusion of contemporary registry data also increases the relevance of the review to current practice, while the sensitivity analyses provide important insight into the robustness of the pooled estimates.

Several limitations should nevertheless be acknowledged. First, the evidence base consisted exclusively of retrospective observational studies, preventing reliable assessment of causality. Second, only two studies reported propensity score-matched or otherwise adjusted data, leaving substantial potential for residual confounding. Third, the imbalance in baseline BMI between the robotic and laparoscopic groups may have contributed to differences in postoperative outcomes, particularly length of stay. Fourth, the included studies varied considerably in patient populations, revisional procedures, institutional practices, surgeon experience, and outcome definitions, contributing to the very high heterogeneity observed for key outcomes. Fifth, the small number of studies prevented formal meta-regression, robust subgroup analyses, and meaningful assessment of publication bias. Finally, all studies were conducted in high-income countries, including the United States, Germany, Switzerland, and Italy, which may limit the generalisability of the findings to other healthcare systems and patient populations.

Taken together, the available evidence suggests that robotic revisional bariatric surgery is a feasible approach that has not been associated with a statistically significant increase in short-term postoperative morbidity compared with laparoscopic surgery. The observed reduction in length of hospital stay is potentially clinically relevant, but the high heterogeneity and sensitivity of this finding to a single large registry-based study mean that it should be interpreted as suggestive rather than definitive. Similarly, the absence of a significant difference in operative time in the primary analysis should not be interpreted as evidence of equivalent operative efficiency, given the very high heterogeneity and substantial changes observed in sensitivity analyses. Overall, the current evidence is insufficient to establish superiority of robotic surgery over laparoscopy in revisional bariatric surgery.

Future research should focus on prospective, multicentre comparative studies with standardised definitions of revisional procedures and perioperative outcomes. Such studies should incorporate robust adjustment for baseline differences and report key potential confounders, including BMI, comorbidity burden, index procedure, indication for revision, surgeon experience, institutional volume, and operative complexity. Procedure-specific reporting would also allow more meaningful comparisons between distinct revisional pathways. Longer-term outcomes, resource utilisation, and cost-effectiveness should additionally be evaluated alongside perioperative outcomes. Until such evidence becomes available, robotic and laparoscopic revisional bariatric surgery should be considered viable approaches, with the choice of technique informed by patient characteristics, procedural complexity, surgeon expertise, institutional resources, and local experience rather than by an assumption of superior outcomes with either platform.

## 5. Limitations

This review addresses key methodological weaknesses in prior meta-analyses through rigorous exclusion of overlapping datasets and structured bias-reduction strategies. However, several important limitations remain. All included studies were retrospective in design, introducing inherent risks of selection bias and unmeasured confounding. Only two of six studies reported propensity score-matched or otherwise adjusted data, limiting our ability to control for baseline differences between robotic and laparoscopic cohorts, including the notable imbalance in mean BMI. Variation in surgical expertise, institutional volume and patient selection criteria represents additional unmeasured confounding that may influence the observed associations. All studies were conducted in high-income countries (United States, Germany, Switzerland, Italy), which may limit generalisability to health systems in low- and middle-income nations. The small number of included studies (*n* = 6) precluded meta-regression to identify sources of heterogeneity and formal statistical assessment of publication bias, which may further confound our findings. Finally, the very high heterogeneity for length of stay and operative time, together with the sensitivity of these results to individual studies, limits the robustness of the conclusions and highlights the need for cautious interpretation.

## 6. Conclusions

In this systematic review and meta-analysis, robotic revisional bariatric surgery was associated with a statistically significant reduction in length of hospital stay compared with laparoscopic surgery, while no significant differences were observed in operative time, reoperation rates or short-term postoperative complications, including leakage and pneumonia. However, the very high statistical heterogeneity, observational design and potential for selection bias mean that these findings should be interpreted as hypothesis-generating rather than definitive evidence of superiority or equivalence. Future prospective, multicentre studies with standardised outcome definitions, procedure-specific reporting and robust adjustment for confounding are needed to clarify the comparative clinical and cost-effectiveness of the two.

## Figures and Tables

**Table 1:** Study Characteristics.

Authors	Publication Year	Country	Study Design	Study Period	Patient Database	Outcomes of Interest
Buchs NC et al. [19]	2013	Switzerland	Retrospective	2000–2013	University Hospital of Geneva	-Length of Procedure -Length of Stay -Reoperation -Total Postoperative Complications
Gray KD et al. [22]	2018	United States	Retrospective	2007–2016	New York Presbyterian Hospital-Weill Cornell Medicine	-Length of Procedure -Length of Stay -Total Postoperative Complications
Beckmann JH et al. [24]	2020	Germany	Retrospective	2013–2015	Referenz-zentrum, German Society of General and Visceral Surgery	-Length of Procedure -Length of Stay -Reoperation -Pneumonia -Total Postoperative Complications
El Chaar M et al. [20]	2020	United States	Retrospective	2015–2017	MBSAQIP	-Length of Procedure
Ugliono E et al. [23]	2022	Italy	Retrospective	2008–2021	University of Turin	-Length of Procedure -Length of Stay -Reoperation -Leakage -Pneumonia -Total Postoperative Complications
Spurzem GF et al. [21]	2024	United States	Retrospective	2015–2022	MBSAQIP	-Length of Stay -Reoperation -Leakage -Pneumonia -Total Postoperative Complications

Abbreviations: MBSAQIP; Metabolic and Bariatric Surgery Accreditation and Quality Improvement Program.

**Table 2:** Baseline Characteristics.

Study	Buchs NC et al.		Gray KD et al. **		Beckmann JH et al.		El Chaar M et al.		Ugliono E et al.		Spurzem GF et al. ***	
Treatment Arm	Robotic	Laparoscopic	Robotic	Laparoscopic	Robotic	Laparoscopic	Robotic	Laparoscopic	Robotic	Laparoscopic	Robotic	Laparoscopic
Population	11	21	18	66	41	18	220	220	150	44	6969	34,435
M/F	1/10	4/17	N/R	N/R	11/30	7/11	35/185	34/186	12/138	11/33	803/6166	4654/29,781
Age *	45.0 ± 9.5	44.0 ± 10.4	N/R	N/R	46.5 ± 10.3	48.8 ± 9.2	49.5 ± 11.3	50.7 ± 11.3	49.0 ± 10.1	48.0 ± 10.5	48.5 ± 10.5	47.4 ± 10.7
BMI *	40.0 ± 8.0	41.0 ± 6.0	N/R	N/R	42.9 ± 8.2	47.1 ± 6.2	42.2 ± 7.6	42.6 ± 8.0	35.6 ± 7.2	38.0 ± 8.5	42.3 ± 8.2	41.4 ± 8.1
Comorbidities												
HTN	2	2	N/R	N/R	N/R	N/R	N/R	N/R	52	18	2928	14,180
Dyslipidaemia	N/R	N/R	N/R	N/R	N/R	N/R	N/R	N/R	6	2	1467	7054
DM	1	1	N/R	N/R	N/R	N/R	N/R	N/R	6	6	575	5565
Sleep Apnoea	3	3	N/R	N/R	N/R	N/R	N/R	N/R	13	4	2016	9813
Index Procedure												
Roux-en-Y	N/R	N/R	N/R	N/R	0	2	N/R	N/R	N/A	N/A	5175	19,148
Sleeve Gastrectomy	N/R	N/R	N/R	N/R	36	13	N/R	N/R	49	5	1794	12,680
Gastric Banding	10	18	N/R	N/R	2	3	N/R	N/R	27	8	N/R	N/R
Gastroplasty	1	3	N/R	N/R	1	0	N/R	N/R	74	31	N/R	N/R

* Continuous data reported in means and SDs. ** Did not present baselines in a [robotic v. laparoscopic] format. *** Spurzem et al. captures all baseline data on account of being the later study. **Abbreviations**: BMI, Body Mass Index; DM, Diabetes Mellitus; HTN, Hypertension; N/R, Not Recorded.

## Data Availability

No new data were created or analyzed in this study. Data sharing is not applicable.

## Supplementary Materials

The following supporting information can be downloaded at https://www.mdpi.com/article/10.3390/medsci14060623/s1, Table S1: Search Strings; Table S2: Risk of Bias Assessment.
