# Supplementary material for: Safety and Efficiency of Robotic Versus Laparoscopic Revisional Bariatric Surgery: A Systematic Review and Meta-Analysis
Source: Med Sci (Basel). 2026 Sep 30;14(6):623. doi: 10.3390/medsci14060623 (PMC13707015; doi:10.3390/medsci14060623)
Supplement: Supplementary file 1 [file medsci-14-00623-s001.zip › medsci-4527530-supplementary.pdf]

SUPPLEMENT

Supplementary Table S1: Search Strings

**Database**	**Search String**
PubMed/ Medline	(“Robotic” OR “robotic surgery” OR “robot” OR “Davinci robot”) AND (“Laparoscopy” OR “laparo scopic bariatric” OR “conventional bariatric”) AND (“revisional surgery” OR “revisional bariatric surgery” OR “secondary bariatric surgery” OR “Conversion”)
Embase	('robotic surgery'/exp OR robot*:ti,ab,kw OR 'davinci robot':ti,ab,kw) AND ('laparoscopy'/exp OR laparoscop*:ti,ab,kw OR 'conventional bariatric':ti,ab,kw) AND ('revisional surgery'/exp OR 'secondary bariatric surgery':ti,ab,kw OR 'conversion surgery':ti,ab,kw OR revisional:ti,ab,kw)
Scopus	(TITLE-ABS-KEY("robotic" OR "robotic surgery" OR "robot" OR "davinci robot")) AND (TITLE-ABS-KEY("laparoscopy" OR "laparoscopic bariatric" OR "conventional bariatric")) AND (TITLE-ABS-KEY("revisional surgery" OR "revisional bariatric surgery" OR "secondary bariatric surgery" OR "conversion"))
Cochrane Central	(“Robotic” OR “robotic surgery” OR “robot” OR “Davinci robot”) AND (“Laparoscopy” OR “laparo scopic bariatric” OR “conventional bariatric”) AND (“revisional surgery” OR “revisional bariatric surgery” OR “secondary bariatric surgery” OR “Conversion”)
Web of Science	TS=("robotic" OR "robotic surgery" OR "robot" OR "davinci robot") AND TS=("laparoscopy" OR "laparoscopic bariatric" OR "conventional bariatric") AND TS=("revisional surgery" OR "revisional bariatric surgery" OR "secondary bariatric surgery" OR "conversion")

Supplementary Table S2: Risk of Bias Assessment

Study ID	Newcastle-Ottawa Scale								
	Selection					*Outcome*			
	Representativeness of Exposed Cohort	*Selection of Non-exposed Cohort*	*Ascertainment of Exposure*	*Outcome not Present at Study Initiation*	*Comparability*	*Assessment of Outcome*	*Follow Up Length*	*Follow Up Adequacy*	*Overall*
Ugliono 2023	*	*	*	*	-	*	*	*	7
Beckmann 2020	*	*	*	*	-	*	*	*	7
Buchs 2013	*	*	*	*	-	*	*	*	7
Spurzem 2024	*	*	*	*	**	*	*	*	9
Elchaar 2020	*	*	*	*	**	*	*	*	9
Gray 2018	*	*	*	*	-	*	*	*	7

★ = star awarded; - = star not awarded. Maximum 4 stars for Selection, 2 for Comparability, 3 for Outcome.
