# Supplementary material for: Safety and Efficiency of Robotic Versus Laparoscopic Revisional Bariatric Surgery: A Systematic Review and Meta-Analysis
Source: Med Sci (Basel). 2026 Sep 30;14(6):623. doi: 10.3390/medsci14060623 (PMC13707015; doi:10.3390/medsci14060623)
Supplement: Supplementary file 1 [file medsci-14-00623-s001.zip › PRISMA_2020_checklist_FILLED.pdf]

PRISMA 2020 Checklist

Section and Topic	Item #	Checklist item	Location where item is reported
**TITLE**			
Title	1	Identify the report as a systematic review.	p. 1 — Title: “A Systematic Review and Meta-Analysis”
**ABSTRACT**			
Abstract	2	See the PRISMA 2020 for Abstracts checklist.	pp. 1–2 — Abstract (structured as Background, Methods, Results and Conclusions; some PRISMA-for-Abstracts elements are not explicitly reported)
**INTRODUCTION**			
Rationale	3	Describe the rationale for the review in the context of existing knowledge.	pp. 2–3 — Introduction: rationale and context; previous reviews and their methodological limitations
Objectives	4	Provide an explicit statement of the objective(s) or question(s) the review addresses.	p. 3 — Introduction: “The present systematic review and meta-analysis therefore aims…”
**METHODS**			
Eligibility criteria	5	Specify the inclusion and exclusion criteria for the review and how studies were grouped for the syntheses.	p. 4, §2.1 Eligibility Criteria — inclusion/exclusion criteria and cohort-overlap rule; grouping for syntheses is not fully explicit
Information sources	6	Specify all databases, registers, websites, organisations, reference lists and other sources searched or consulted to identify studies. Specify the date when each source was last searched or consulted.	p. 4, §2.2 Literature Search Process — PubMed/Medline, Cochrane Central, Embase, Scopus, Web of Science; searched to 12 Dec 2024; reference lists also searched
Search strategy	7	Present the full search strategies for all databases, registers and websites, including any filters and limits used.	p. 4, §2.2; Supplementary Table 1 — full strategies are stated to be provided there, but the supplied manuscript does not display the actual strategies
Selection process	8	Specify the methods used to decide whether a study met the inclusion criteria of the review, including how many reviewers screened each record and each report retrieved, whether they worked independently, and if applicable, details of automation tools used in the process.	p. 4, §2.2 — two reviewers independently screened titles/abstracts/full texts; EndNote used for duplicate removal; third reviewer resolved discrepancies
Data collection process	9	Specify the methods used to collect data from reports, including how many reviewers collected data from each report, whether they worked independently, any processes for obtaining or confirming data from study investigators, and if applicable, details of automation tools used in the process.	p. 4, §2.3 — standardised Excel extraction described; number/independence of data extractors and investigator contact procedures are not explicitly stated
Data items	10a	List and define all outcomes for which data were sought. Specify whether all results that were compatible with each outcome domain in each study were sought (e.g. for all measures, time points, analyses), and if not, the methods used to decide which results to collect.	p. 4, §2.3 — six outcomes listed; selection of all compatible measures/time points/results is not explicitly described
	10b	List and define all other variables for which data were sought (e.g. participant and intervention characteristics, funding sources). Describe any assumptions made about any missing or unclear information.	pp. 4, 7–8, §2.3 and Table 2 — baseline/participant characteristics reported; full list of other sought variables and assumptions for missing/unclear data are not explicitly stated
Study risk of bias assessment	11	Specify the methods used to assess risk of bias in the included studies, including details of the tool(s) used, how many reviewers assessed each study and whether they worked independently, and if applicable, details of automation tools used in the process.	p. 4, §2.3 — Newcastle–Ottawa Scale; two independent reviewers; discrepancies resolved through discussion
Effect measures	12	Specify for each outcome the effect measure(s) (e.g. risk ratio, mean difference) used in the synthesis or presentation of results.	p. 5, §2.4 — MD for continuous outcomes and OR/RR methods for dichotomous outcomes. Note: the text initially states RR, whereas pooled results are reported as ORs
Synthesis methods	13a	Describe the processes used to decide which studies were eligible for each synthesis (e.g. tabulating the study intervention characteristics and comparing against the planned groups for each synthesis (item #5)).	pp. 4–5, §§2.1–2.4; pp. 5–10 Results — eligible comparative studies and outcome-specific contributing studies identified; process for assigning studies to each synthesis is only partly

PRISMA 2020 Checklist

Section and Topic	Item #	Checklist item	Location where item is reported
			explicit
	13b	Describe any methods required to prepare the data for presentation or synthesis, such as handling of missing summary statistics, or data conversions.	p. 5, §2.4 — median/IQR conversion to mean/SD; averaging continuous data and summing dichotomous data; log-transformation of ORs
	13c	Describe any methods used to tabulate or visually display results of individual studies and syntheses.	pp. 6–10, Tables 1–2 and Figures 2–7 — study characteristics/baselines tabulated and meta-analysis results shown in forest plots
	13d	Describe any methods used to synthesize results and provide a rationale for the choice(s). If meta-analysis was performed, describe the model(s), method(s) to identify the presence and extent of statistical heterogeneity, and software package(s) used.	p. 5, §2.4 — Review Manager 5.4.1; random-effects model; inverse-variance methods; I^2 heterogeneity assessment
	13e	Describe any methods used to explore possible causes of heterogeneity among study results (e.g. subgroup analysis, meta-regression).	p. 5, §2.4 — sensitivity analysis to investigate heterogeneity; subgroup analysis planned where ≥3 studies were available; meta-regression/publication-bias assessment planned only if >10 studies
	13f	Describe any sensitivity analyses conducted to assess robustness of the synthesized results.	p. 5, §2.4 — leave-one-out sensitivity analysis planned for all outcomes
Reporting bias assessment	14	Describe any methods used to assess risk of bias due to missing results in a synthesis (arising from reporting biases).	p. 5, §2.4 — publication-bias assessment planned only if >10 studies reported an outcome; no formal assessment was performed with six studies
Certainty assessment	15	Describe any methods used to assess certainty (or confidence) in the body of evidence for an outcome.	Not reported — no formal certainty/confidence assessment method (e.g. GRADE) is described
**RESULTS**			
Study selection	16a	Describe the results of the search and selection process, from the number of records identified in the search to the number of studies included in the review, ideally using a flow diagram.	pp. 5–6, §3.1 and Figure 1 — 2093 unique records identified and 6 studies included; PRISMA flowchart provided
	16b	Cite studies that might appear to meet the inclusion criteria, but which were excluded, and explain why they were excluded.	Not reported — excluded studies are not individually cited with reasons; only aggregate exclusion reasons/counts are shown in Figure 1
Study characteristics	17	Cite each included study and present its characteristics.	pp. 6–8, §3.2 and Table 1 — all six included studies cited and study characteristics presented
Risk of bias in studies	18	Present assessments of risk of bias for each included study.	p. 7, §3.3 and Supplementary Table 2 — Newcastle–Ottawa assessments reported for each included study
Results of individual studies	19	For all outcomes, present, for each study: (a) summary statistics for each group (where appropriate) and (b) an effect estimate and its precision (e.g. confidence/credible interval), ideally using structured tables or plots.	pp. 8–10, §§3.5.1–3.5.6 and Figures 2–7 — individual-study estimates are shown in forest plots; structured per-study summary statistics are not presented separately for every outcome
Results of syntheses	20a	For each synthesis, briefly summarise the characteristics and risk of bias among contributing studies.	pp. 6–8, §§3.2–3.4 and Supplementary Table 2 — study characteristics, baseline data and risk of bias summarised
	20b	Present results of all statistical syntheses conducted. If meta-analysis was done, present for each the summary estimate and its precision (e.g. confidence/credible interval) and measures of statistical heterogeneity. If comparing groups, describe the direction of the effect.	pp. 8–10, §§3.5.1–3.5.6 and Figures 2–7 — pooled estimates, 95% CIs, p-values and I^2 reported for all six outcomes
	20c	Present results of all investigations of possible causes of heterogeneity among study results.	pp. 5 and 8–12 — sensitivity analyses investigate influential studies/heterogeneity; no formal subgroup/meta-regression analysis was performed because of the small number of studies
	20d	Present results of all sensitivity analyses conducted to assess the robustness of the synthesized results.	pp. 8–10, §§3.5.1–3.5.6 — leave-one-

PRISMA 2020 Checklist

Section and Topic	Item #	Checklist item	Location where item is reported
			out/influential-study sensitivity analyses reported where feasible
Reporting biases	21	Present assessments of risk of bias due to missing results (arising from reporting biases) for each synthesis assessed.	Not reported — no formal assessment of risk of bias due to missing results/publication bias was performed
Certainty of evidence	22	Present assessments of certainty (or confidence) in the body of evidence for each outcome assessed.	Not reported — no formal certainty assessment of the body of evidence is presented
**DISCUSSION**			
Discussion	23a	Provide a general interpretation of the results in the context of other evidence.	pp. 10–14, §4 Discussion — results interpreted in relation to previous meta-analyses and methodological limitations
	23b	Discuss any limitations of the evidence included in the review.	pp. 13–14, §5 Limitations and Discussion — retrospective design, confounding, BMI imbalance, heterogeneity, small study number and generalisability discussed
	23c	Discuss any limitations of the review processes used.	pp. 13–14, §4 Discussion and §5 Limitations — limitations of the review process, including overlap, confounding, heterogeneity and inability to perform some analyses
	23d	Discuss implications of the results for practice, policy, and future research.	pp. 14, §4 Discussion/§6 Conclusions — implications for practice and recommendations for prospective multicentre studies, standardised definitions and cost-effectiveness research
**OTHER INFORMATION**			
Registration and protocol	24a	Provide registration information for the review, including register name and registration number, or state that the review was not registered.	Not reported — no registration name or registration number is provided
	24b	Indicate where the review protocol can be accessed, or state that a protocol was not prepared.	Not reported — no accessible protocol or statement that no protocol was prepared is provided
	24c	Describe and explain any amendments to information provided at registration or in the protocol.	Not reported — no amendments to registration/protocol are described
Support	25	Describe sources of financial or non-financial support for the review, and the role of the funders or sponsors in the review.	Not reported — no source of financial/non-financial support or funder role is stated
Competing interests	26	Declare any competing interests of review authors.	Not reported — no explicit competing-interest declaration is included in the supplied manuscript
Availability of data, code and other materials	27	Report which of the following are publicly available and where they can be found: template data collection forms; data extracted from included studies; data used for all analyses; analytic code; any other materials used in the review.	p. 14, Supplementary Materials — supplementary items are listed, but public availability of the extraction dataset, analysis dataset, analytic code and other review materials is not explicitly stated

From: Page MJ, McKenzie JE, Bossuyt PM, Boutron I, Hoffmann TC, Mulrow CD, et al. The PRISMA 2020 statement: an updated guideline for reporting systematic reviews. BMJ 2021;372:n71. doi: 10.1136/bmj.n71. This work is licensed under CC BY 4.0. To view a copy of this license, visit https://creativecommons.org/licenses/by/4.0/

Filled from the supplied systematic review manuscript. “Not reported” indicates that the manuscript does not contain the information required by that PRISMA item; “partial” notes where the item is only incompletely addressed.

PRISMA

PRISMA 2020 Checklist

Key PRISMA items to consider revising in the manuscript: 7 (full search strategies), 9–10 (data extraction/details of variables), 14–15 (reporting bias and certainty), 16b (excluded studies), 19 (per-study outcome data), 21–22 (reporting bias/certainty results), and 24–27 (registration/protocol, support, competing interests, and data/code availability).
